# Genetic Determinants of Progressive Pulmonary Fibrosis: A Comprehensive Review

**DOI:** 10.3390/ijms262411846

**Published:** 2025-12-08

**Authors:** Ardak Zhumagaliyeva, Joanna Chorostowska-Wynimko, Aleksandra Jezela-Stanek

**Affiliations:** 1 Department of Family Medicine No.2, Astana Medical University, Astana 010000, Kazakhstan; ardak.zhumagalieva77@gmail.com; 2 Department of Genetics and Clinical Immunology, National Institute of Tuberculosis and Lung Diseases, 01-138 Warsaw, Poland; jezela@gmail.com

**Keywords:** progressive pulmonary fibrosis, idiopathic pulmonary fibrosis, genetic variants, telomere biology genes, surfactant protein genes, *MUC5B* promoter, epigenetics, precision medicine

## Abstract

Progressive pulmonary fibrosis (PPF) encompasses fibrosing interstitial lung diseases marked by relentless scarring of the lungs, leading to respiratory failure. Although its pathogenesis remains incompletely understood, recent genetic discoveries have shed light on the molecular mechanisms that drive PPF onset and progression. This comprehensive review summarizes current knowledge of PPF genetics, highlighting both rare pathogenic variants and more common susceptibility polymorphisms. Key genetic contributors include telomere maintenance genes, surfactant protein genes, and the *MUC5B* promoter variant rs35705950, which is the strongest known genetic risk factor for idiopathic pulmonary fibrosis. We also discuss epigenetic factors such as DNA methylation and histone modifications that regulate fibrotic gene expression. Integrating genetic findings with clinical phenotypes reveals distinct disease endotypes with different prognoses and therapeutic responses, laying the groundwork for precision medicine in PPF treatment. Finally, we address the clinical implications of PPF genetics, including advances in genetic testing, biomarker development, and emerging gene-targeted treatment strategies.

## 1. Introduction

Progressive pulmonary fibrosis (PPF) encompasses a spectrum of interstitial lung diseases characterized by the progressive accumulation of scar tissue in the lungs, leading to the irreversible loss of lung function and ultimately respiratory failure [1,2]. Idiopathic pulmonary fibrosis (IPF), the most common idiopathic form falling under PPF umbrella, is a rare disease with an estimated prevalence in the United States of 27.2 per 100,000 individuals (approximately 0.027% of the population) [3]. It predominantly affects the elderly, and its prevalence is increasing globally as populations age. Despite the introduction of antifibrotic therapies, the prognosis remains serious, with a median survival from diagnosis of 3 to 5 years in untreated patients, although modern treatments can modify this trajectory [4,5].

Given this serious outlook, genetic predisposition has emerged as a crucial contributor to PPF pathogenesis alongside environmental exposures and aging-related mechanisms [4,6]. Although pulmonary fibrosis was historically regarded as a sporadic disease, increasing evidence substantiates the substantial contribution of genetic factors to both disease onset and progression. Familial clustering is observed in up to 20% of patients with idiopathic pulmonary fibrosis, and these familial forms—referred to as familial pulmonary fibrosis (FPF)—typically follow an autosomal dominant inheritance pattern with incomplete penetrance [7,8].

Concurrently, advances in genomic technologies have transformed our understanding of PPF genetics. Large-scale genome-wide association studies (GWASs) have identified numerous loci associated with disease susceptibility, while whole-exome and whole-genome sequencing approaches have uncovered rare pathogenic variants across selective gene families [4,9]. Collectively, these discoveries highlight telomere biology, surfactant protein metabolism, and mucociliary clearance as pivotal biological pathways in PPF pathogenesis, providing the foundation for increasingly precise genetic and mechanistic insights into fibrotic lung disease.

## 2. Genetic Architecture of Progressive Pulmonary Fibrosis

Pulmonary fibrosis’s genetic architecture is a mixture of common and rare variants, with telomere biology and mucin regulation playing critical roles. Among the best-replicated and most significant common-variant associations is polymorphism in the *MUC5B* promoter (rs35705950), which substantially increases the risk of fibrotic interstitial lung disease, including IPF [10,11]. Rare telomere-maintenance gene pathogenic variants, such as *TERT*, *TERC*, *RTEL1*, and *PARN*, are principal causes of pulmonary fibrosis and familial pulmonary fibrosis and are associated with shorter telomeres, earlier disease onset, and poor outcomes [10,12,13].

The comprehensive review by Adegunsoye et al. stresses that susceptibility is not a monogenic trait but exhibits a polygenic and multifactorial inheritance pattern, with numerous variants of moderate effect acting in concert with rare high-effect alleles and environmental exposures [10]. The authors highlight gene–environment interactions, like smoking, air pollution, and exogenous exposures, as determinants of disease risk and course. They also highlight the absence of non-European populations in current genomic investigations, with a future direction towards increased inclusion to more appropriately reflect global genetic heterogeneity.

Prospects consist of integration of genomic data with additional omics layers (transcriptomics, epigenomics, and proteomics) and the development of polygenic risk scores (PRSs) as potential future routes toward more refined risk stratification, potentially informing precision medicine approaches and assisting in the establishment of disease classification within the progressive pulmonary fibrosis spectrum.

The largest known genetic-predisposing variant is the *MUC5B* promoter variant rs35705950, with an estimated six-fold excess risk of developing idiopathic pulmonary fibrosis (IPF) in heterozygotes and twenty-fold excess risk in homozygotes [11,14]. The variant has the unique relationship of having high disease susceptibility with improved survival, an association reliably recapitulated in follow-up studies.

Other variant alleles collectively define disease susceptibility with smaller individual effects. Genome-wide association has focused attention on loci in host-defense-related genes, epithelial-integrity-related genes, and cell–matrix interaction-related genes, including *DSP*, *FAM13A*, *TOLLIP*, *SPPL2C*, and *ATP11A*, together with *MUC5B*, explaining a considerable amount of the transmitted risk [1,10].

In contrast, rare pathogenic variants, most notable in telomere-maintenance genes such as *TERT*, *TERC*, *RTEL1*, and *PARN*, display larger effect sizes and strong association with short telomeres, premature disease onset, and poor clinical course [10,12,13]. These rare alleles, with a large effect, have joint background common variants and environmental exposures (e.g., smoking, environmental exposures, work-related dust), producing the complex genetic architecture of progressive pulmonary fibrosis.

Notably, variants in telomere-maintenance genes (e.g., *TERT*, *TERC*, *PARN*, *RTEL1*) are often loss-of-function variants (nonsense or frameshift changes that shorten telomeres), whereas variants in surfactant protein genes (e.g., *SFTPC*, *SFTPA2*, *ABCA3*) are frequently missense changes that disrupt protein folding or function [15]. In contrast, the mucociliary clearance risk factor *(MUC5B* rs35705950) is a non-coding promoter polymorphism that increases gene expression, rather than being a coding mutation.

All the variants discussed in this review are single-nucleotide variants (SNVs) or small insertions/deletions in specific genes. To our knowledge, no recurrent large structural variants (such as major chromosomal deletions or duplications) have been definitively linked to progressive pulmonary fibrosis; the genetic risk is predominately conferred by point mutations in the genes highlighted, but, as larger structural variants lead to greater disruption in gene functions, their impact in this area is also feasible.

Common genetic variants, typically defined as those with a minor allele frequency (MAF) greater than 5% in the general population, individually confer modest increases in the risk of pulmonary fibrosis, but their cumulative effects can be substantial [10].

### 2.1. MUC5B Promoter Variant

The best-characterized and most prominent genetic risk variant of PPF is the gain-of-function promoter variant rs35705950 of the *MUC5B* gene [11,14]. This single-nucleotide polymorphism, located approximately 3 kb upstream of the transcriptional start site and near an FOXA2 binding site, is an effective cis-regulator, eQTL, which leads to ~34-fold increases in *MUC5B* expression in healthy lung and ~5-fold increases in IPF lungs [11]. It is posited to act in part through an interaction with the transcription factor XBP1s of the unfolded protein response, linking it with endoplasmic reticular stress pathways [4]. Immunohistochemical interrogations disclose dense protein accumulations of MUC5B in terminal bronchioles and cysts of honeycombs, supporting its distal airway pathologic role [11].

T-allele prevalence is approximately 35% in patients with IPF compared with ~9% in healthy controls; hence, a strong common genetic background to fibrotic lung disease is transmitted by rs35705950 [11]. Notably, there is evident population stratification of the variant: the variant is common among individuals with European ancestry but is rare in African and East Asian populations, and it mirrors worldwide IPF prevalence disparities [11]. The rs35705950 variant is strongly associated with increased susceptibility to pulmonary fibrosis, though it is not fully penetrant (many carriers do not develop disease).

Clinically, variant rs35705950 has the seeming contradiction of having increased disease risk and improved survival. Carriers have considerably longer survival and slower progression of lung function decline than non-carriers, with results reproduced in multiple cohorts, including those with antifibrotic therapy [16,17,18]. These effects, hypothetically, indicate a lower microbial burden and perhaps a different pathobiological endotype. There does, however, appear population-specific variability: an illustration of this can be found in a Chinese cohort with an inverse correlation, with T allele having lower FVC (Forced Vital Capacity) and DL_CO_ (Diffusing Capacity of the Lungs for Carbon Monoxide) and higher mortality [19]. Accordingly, studies in Chinese cohorts have found either a much weaker association or no significant association of *MUC5B* with pulmonary fibrosis, which is likely due to the low allele frequency found in Asian populations [15]. This population difference may partly explain the lower incidence of IPF in East Asian populations relative to European populations.

### 2.2. Additional GWAS-Identified Loci

Beyond *MUC5B*, multiple GWASs have identified over 20 additional genetic loci associated with PPF susceptibility [4,20]. These loci implicate a diverse range of biological pathways.

### 2.3. Telomere Maintenance Genes

Telomere biology is the main determinant of susceptibility, onset, and disease course in idiopathic pulmonary fibrosis and the other PPF forms. Common alleles in the telomere maintenance genes *TERT*, *TERC*, and *RTEL1* were discovered to be connected with disease susceptibility and inter-individual differences in leukocyte telomere length [4,21]. Notably, common susceptibility alleles have effects that tend to differ from those of rare, high-impact alleles in the same genes, implicating complex gene-dosage and regulatory mechanisms.

Rare heterozygous loss-of-function mutations in *TERT*, *TERC*, *RTEL1*, *PARN* (as discussed below), and other telomere genes are found in 20–30% of familial pulmonary fibrosis and in 2–5% of apparent sporadic pulmonary fibrosis [12,13]. Such pathogenic mutations cause abrupt telomere shortness, so clinical disease manifests earlier, clinical disease is more virulent, bone marrow failure occurs, and liver disease develops. In contrast, common polymorphisms in *TERT*, *TERC*, or *RTEL1* tend to have smaller effect sizes and do not necessarily diminish telomeres to the same degree; indeed, some are seen to correlate with slightly longer telomeres, implicating minute changes in telomere homeostasis but no frank loss of function in communicating genetic susceptibility to pulmonary fibrosis [10].

Mechanistically, telomere shortening causes alveolar epithelial cell senescence, apoptosis, and dysfunctional regenerative responses, triggering profibrotic signaling through TGF-β activation and DNA damage responses. Reduced transplant-free survival has been correlated with shortened telomeres, and telomere length has been confirmed as a potential prognostic biomarker and therapeutic target. Encouraging early clinical trials of telomerase activators and telomere end-stabilizing agents are also being tested and reflect the translational potential of these pathways [10,13].

Overall, these findings unveil a spectrum of telomere-related risk, from mild common variants with moderately sized telomere dynamic effects to rare, high-penetrance mutations with instant compromise of telomere integrity, collectively shaping the genetic architecture and clinical phenotypes of progressive pulmonary fibrosis.

### 2.4. Cell Adhesion and Signaling

*DSP* (desmoplakin), *FAM13A*, and *TOLLIP* variants impact intercellular adhesion and inflammatory signaling pathways [4,21]. The DNA variant *DSP* rs2076295 has been associated with heightened disease risk and altered DNA methylation profiles, evidencing epigenetic regulatory mechanisms [22]. In an IPF patient cohort from the Czech group of the EMPIRE registry, IPF patients carrying the *DSP* rs2076295 G allele were found to have better clinical responses to nintedanib, with longer overall survival and a slower rate of lung function decline compared with TT genotype carriers, while TT homozygotes benefited most from pirfenidone [23].

Bonella et al. indicated that IPF patients who were heterozygous or homozygous carriers of the minor allele of *TOLLIP* rs5743890 had poor outcomes with approximately 20 months’ median overall survival reduction. There was also greater FVC decline in heterozygous and homozygous carriers, which indicates that rs5743890 may also serve as a genetic biomarker of disease progression and IPF risk stratification [24]. Variant *TOLLIP* rs3750920 has been correlated with response to treatment, with Oldham et al. showing carriers having improved results with N-acetylcysteine during the pre-antifibrotic era, pointing to a pharmacogenetic interaction [25]. There is also a variant correlation with imbalanced lung microbiota, pointing to a potential association with dysfunctional innate immunity and microbial environment influencing disease course [26].

A meta-analysis identified the G allele of *FAM13A* rs2609255 variant to have an important role in susceptibility to interstitial lung diseases across populations, with increased association also identified in IPF in Asian, non-Hispanic American, and Hispanic white populations and European populations with rheumatoid-arthritis-related ILD [27]. For IPF, *FAM13A* polymorphisms such as rs2609255 have been posited as possible clinical predictors of prognosis, but gene expression and clinical relevance studies to date are lacking [27].

The risk variant in *DSP* lies in a non-coding region and has been associated with altered DSP expression in fibrotic lung tissue [28], suggesting a functional regulatory impact. Similarly, the IPF-associated SNP in TOLLIP (Toll interacting protein)—notably, rs5743890—correlates with lower *TOLLIP* gene expression and was linked to worse survival in IPF patients carrying the minor allele [28], indicating a functional consequence. In contrast, variants identified near *FAM13A*, *SPPL2C*, and *ATP11A* through GWAS are common polymorphisms with modest effect sizes. Their direct functional effects remain less clear, although they implicate important pathways (e.g., *FAM13A* is thought to influence Wnt signaling in lung cells, and the *SPPL2C* locus at 17q21.31 is within a complex inversion haplotype associated with multiple lung traits [28]). Ongoing research is evaluating whether these variants alter gene expression or protein function.

### 2.5. Mitotic Spindle Assembly

Recent discoveries have implicated *KIF15* and *MAD1L1* in PPF susceptibility, suggesting roles for cell cycle regulation and genomic stability in disease pathogenesis [29]. These findings highlight novel pathways that may be amenable to therapeutic targeting.

Rare missense and deleterious variants in *KIF15* are enriched in individuals with IPF, suggesting a functional link to disease mechanisms [30]. In a cohort of approximately 138 patients, carriers of *KIF15* missense variants were diagnosed at a significantly younger median age of 54 years compared with 72 years in non-carriers (*p* = 0.023) and showed higher rates of early lung transplantation or mortality, highlighting *KIF15* as a potential prognostic biomarker for early-onset and severe IPF [30].

Although *MAD1L1* has not been directly associated with specific clinical phenotypes such as age at onset or survival, its consistent identification as an IPF risk locus across independent cohorts indicates a likely role in disease pathogenesis [15]. Unlike other IPF-associated loci, *MAD1L1* has not yet been systematically studied for genotype–phenotype correlations.

### 2.6. mTOR Signaling

The finding that *DEPTOR* is a PPF susceptibility gene is consistent with the significant role of mTOR signaling in lung fibrosis [20]. Reduced *DEPTOR* expression has been found to correlate with increased disease risk, a finding also in keeping with experimental data implicating activated mTOR in fibrotic processes.

Although there have been no developed direct correlations between *DEPTOR* gene variants per se and clinical endpoints such as survival or disease progression, there have been notable imaging–genetic results. In a study performed by Mohammadi-Nejad et al., the authors demonstrated that the IPF-related risk variant for *DEPTOR* is associated with structural changes in the brain, with an association with thinning in the anterior cingulate cortex and overall changes in white matter integrity [31]. These associations appear to be explained in part through pulmonary function tests, with FVC specifically having notable correlations with these associations. These findings suggest an effect of *DEPTOR* variation systematically outside of the lung and provide a potential explanation for the comorbidities such as neuropsychiatric issues (e.g., depression, anxiety) seen in IPF patients [20,31].

The genes implicated in progressive pulmonary fibrosis converge on several key biological pathways that collectively shape disease susceptibility and progression. These include pathways involved in telomere maintenance, where loss-of-function variants lead to accelerated cellular senescence; surfactant metabolism, in which misfolded proteins drive endoplasmic reticulum stress and epithelial injury; mucociliary clearance, highlighted by the *MUC5B* promoter variant that alters airway mucus biology; cell adhesion and innate immune signaling, where variants in *DSP*, *TOLLIP*, and *FAM13A* modulate epithelial stability and host responses; and mitotic spindle or mTOR signaling, which are pathways increasingly recognized as contributors to aberrant tissue repair and fibroblast activation. Rare variants causing syndromic disorders, such as Hermansky–Pudlak syndrome or telomere biology syndromes, further demonstrate how defects in lysosome-related organelles or telomerase function can culminate in aggressively progressive fibrosis. Table 1 summarizes these major pathways and the representative genes, variant types, and clinical implications associated with each.

### 2.7. Rare Genetic Variants

While common variants account for the majority of genetic risk in sporadic PPF, rare pathogenic variants with high penetrance are responsible for most cases of familial disease [8,32]. These variants, typically with minor allele frequencies less than 0.1%, are found in approximately 25% of FPF families and can also occur in apparently sporadic cases.

### 2.8. Telomere-Related Genes

The first demonstration that telomerase mutations cause adult-onset familial pulmonary fibrosis was provided by Tsakiri et al., who identified heterozygous *TERT* and *TERC* variants in multiple affected families [33]. Variants in genes involved in telomere maintenance represent the most common cause of monogenic PPF, accounting for approximately 15–20% of familial cases [7,34]. The major telomere-related genes involved in PPF pathogenesis are *TERT*, *TERC*, *RTEL1*, and *PARN.*

*TERT* (telomerase reverse transcriptase): Variants in *TERT* result in reduced telomerase activity and accelerated telomere shortening [33,34]. These variants demonstrate autosomal dominant inheritance with incomplete penetrance and are associated with premature aging phenotypes affecting multiple organ systems.

The penetrance of pulmonary fibrosis in *TERT* variant carriers increases with age; it is rare before 40 years but affects about 60% of men and 50% of women over 60 years [35]. Environmental exposures, particularly cigarette smoke and inhaled fibrogenic agents, markedly elevate disease risk [35,36]. Most affected individuals are diagnosed with idiopathic pulmonary fibrosis, with approximately 74% showing UIP on CT and about 86% demonstrating UIP histologically [35]. In this group, pulmonary fibrosis often progresses rapidly, with a mean survival of about three years after diagnosis. The average age at death is approximately 58 years in men and 67 years in women, both lower than population norms [35]. In a cohort of 237 patients, carriers of *TERT* or *TERC* disease-associated variants had a median transplant-free survival of about 4.2 years, compared with 7.2 years in non-carriers [37]. Extrapulmonary features in *TERT* variant carriers may include macrocytosis, thrombocytopenia, mild cytopenias, premature hair greying, liver disease, and bone marrow dysfunction, consistent with telomere syndromes [8,37].

*TERC* (telomerase RNA component): *TERC* variants affect the RNA template component of telomerase, leading to similar cellular phenotypes as *TERT* variants [33,34]. Affected individuals often develop bone marrow failure, liver disease, and other telomere syndrome manifestations in addition to pulmonary fibrosis.

Compared with other telomere-related gene variants, pulmonary fibrosis associated with *TERC* variants typically presents earlier, with a mean onset of about 51 ± 11 years [38]. At diagnosis, a usual interstitial pneumonia pattern on HRCT is seen in about two-thirds of individuals with *TERT* or *TERC* variants, compared with fewer than 7% of those without telomerase-related mutations [39]. Longitudinal imaging shows that patients with atypical baseline patterns often progress to a classic UIP appearance, reflecting the progressive nature of disease across telomerase variant subtypes [38]. Hematologic abnormalities are frequent in *TERC* variant carriers and commonly include macrocytosis, thrombocytopenia, and mild cytopenias [37]. Newton et al. reported that telomerase-related variant carriers, including *TERC*, experience a mean annual FVC decline of about 300 mL and a 5.8% drop in predicted DL_CO_, with a median interval from diagnosis to death or lung transplantation of approximately 2.9 years [38].

*PARN* (Poly(A)-Specific Ribonuclease): Originally discovered through exome sequencing of familial cases, *PARN* variants account for approximately 2–3% of FPF families [12]. *PARN* functions in telomerase biogenesis and RNA processing, linking telomere biology to post-transcriptional gene regulation.

A multicenter retrospective study found that about 74% of individuals had a smoking history or exposure to fibrogenic agents such as occupational dust, suggesting a substantial role of environmental factors in disease susceptibility and clinical expression [15,40]. Carriers of mutations in *TERT*, *RTEL1*, *PARN*, and *TERC* consistently exhibit progressive decline in absolute FVC, indicating that telomere-related variants predict disease progression irrespective of the underlying clinical diagnosis [15]. Time to death or lung transplantation was similar among individuals with *PARN*, *TERT*, or *TERC* variants, indicating comparable prognostic outcomes for these telomerase-related variants [38]. Extrapulmonary manifestations were less common in *PARN* variant carriers than in those with *TERC* or *TERT* mutations [40].

*RTEL1* (regulator of telomere elongation helicase 1): *RTEL1* variants affect telomere replication and stability, contributing to approximately 6% of FPF families [12,33]. The protein functions as a DNA helicase involved in telomere maintenance and DNA repair processes.

Additional telomere-related genes including *DKC1*, *TINF2*, *NAF1*, and *ZCCHC8* have been associated with PPF in smaller numbers of families [7,41].

The *RTEL1* variants are most frequently associated with IPF, although other pulmonary phenotypes may also occur [33]. In patients with different telomere-related gene variants, the mean telomere length was shortest in *TERC* carriers, followed by *TERT*, *RTEL1*, and *PARN.* Extrapulmonary manifestations, particularly hematologic and hepatic abnormalities, occur but are less frequent than in other telomere-related disorders, with mean age at ILD diagnosis following the same sequence. Individuals with *RTEL1* variants were diagnosed at a mean age of 60 years [34,38]. The phenotypic heterogeneity of *RTEL1*-associated interstitial lung disease underscores the need for thorough clinical evaluation to guide individualized management [42].

### 2.9. Surfactant Protein Genes

Variants in genes encoding surfactant proteins account for approximately 1–3% of PPF cases but are associated with distinct clinical features including early-onset disease and co-occurrence with lung cancer [7,8].

*SFTPC* (surfactant protein C): *SFTPC* variants were among the first genetic causes of PPF to be identified [8,43]. These mutations result in misfolded proteins that trigger endoplasmic reticulum stress and unfolded protein response pathways. Affected individuals can present with disease onset ranging from infancy to late adulthood within the same family.

In individuals with *SFTPC* variants, histopathologic findings include NSIP, DIP, chronic pneumonitis of infancy, and UIP, while imaging commonly reveals ground-glass opacities, reticulation, and honeycombing [44]. Clinical manifestations are not reliably predicted by genotype due to incomplete penetrance and variable expressivity. The I73T variant is the most frequent pathogenic form and occurs across age groups and histologic patterns, whereas variants such as G100S are associated with familial clustering [44,45]. In surfactant-related disease, infants may present with rapidly progressing illness, whereas adults typically experience gradual fibrotic progression culminating in respiratory failure. Some adults advance to end-stage disease requiring transplantation, with prognosis influenced by age at onset, specific variant, coexisting genetic modifiers, and environmental factors. Prognostic estimates remain uncertain due to small cohort sizes and ascertainment bias [46,47].

*SFTPA1* and *SFTPA2* (surfactant proteins A1 and A2): Variants in these nearly identical genes are associated with both PPF and lung adenocarcinoma [47,48]. The proteins function as collectins, involved in innate immunity and surfactant homeostasis. Affected families require enhanced lung cancer surveillance protocols.

Affected individuals demonstrate considerable clinical, radiologic, and histopathologic heterogeneity. Imaging may reveal UIP with ground-glass opacities, reticulation, and subpleural fibrosis, or nonspecific and mixed patterns. Histology ranges from UIP and NSIP to other chronic interstitial pneumonias. Genotype does not reliably predict onset or progression, which are modulated by environmental and additional genetic factors [48].

*ABCA3* (ATP-Binding Cassette Transporter A3): *ABCA3* variants typically cause autosomal recessive surfactant deficiency disorders [8]. While most are commonly associated with neonatal respiratory distress, some variants can cause adult-onset pulmonary fibrosis.

Li et al. reported that ILD associated with *ABCA3* variants progresses over time, with a mean absolute FVC decline of 1.1% per year and increasing cystic changes on serial CT in patients with repeated imaging [49]. Histopathologic patterns vary and include chronic pneumonitis of infancy, nonspecific interstitial pneumonia, and desquamative interstitial pneumonia [49]. Family and single-case reports show that identical variants may produce markedly different phenotypes within the same family [50]. Case series and cohort studies have identified *ABCA3* variants in adults with ILD, with phenotypes overlapping those of *SFTPC*-related disease and ranging from NSIP to UIP. Combined pulmonary fibrosis and emphysema have been reported, particularly in smokers. Age at onset and disease progression are highly variable, and current evidence does not allow genotype-based prediction of clinical course [51].

### 2.10. Hermansky–Pudlak Syndrome

Hermansky–Pudlak syndrome (HPS) represents a unique form of genetic PPF caused by variants in genes affecting lysosome-related organelles [6,52]. HPS is characterized by oculocutaneous albinism, bleeding diathesis, and, in certain subtypes, progressive pulmonary fibrosis. The syndrome is particularly common in Puerto Rico due to founder variants in *HPS1* and *HPS3* genes. HPS-associated pulmonary fibrosis typically develops in the third–fourth decades of life and follows a more aggressive clinical course than typical IPF.

Pulmonary fibrosis occurs most frequently in Hermansky–Pudlak syndrome (HPS) types 1 and 4, caused by BLOC-3 complex deficiency, and less often in HPS-2 due to AP-3 deficiency [53]. Nearly all individuals with HPS-1 develop HPS-associated pulmonary fibrosis, as in IPF [52]. Clinical features of HPS-PF resemble those of IPF, although onset usually occurs at 30 to 40 years in HPS-PF and after 50 years in IPF [52]. Symptoms generally arise in the fourth decade in HPS-1, in middle adulthood in HPS-4, and in childhood or early adulthood in HPS-2 [54,55,56]. HPS-2 is additionally associated with recurrent infections, neutropenia responsive to granulocyte colony-stimulating factor, and occasional spontaneous pneumothorax [55]. High-resolution CT commonly shows a UIP pattern with basal-predominant reticulation, traction bronchiectasis, and honeycombing, while histopathology often confirms UIP, though NSIP and mixed patterns may occur [57].

Beyond DNA sequence variants, epigenetic modifications further influence gene expression in PPF, as discussed next.

## 3. Epigenetic Regulation

### 3.1. Epigenetic Modifications

Beyond genetic sequence variations, epigenetic modifications play crucial roles in PPF pathogenesis by regulating gene expression patterns without altering DNA sequence [58,59]. These modifications include DNA methylation, histone modifications, and non-coding RNA regulation, all of which contribute to the complex molecular landscape of fibrotic lung disease.

### 3.2. DNA Methylation

Genome-wide DNA methylation profiling has revealed extensive methylation changes in PPF lung tissue, with over 2000 differentially methylated regions identified compared to healthy controls [59]. These methylation changes are enriched in CpG island shores and demonstrate significant correlations with gene expression alterations. Importantly, 60% of differentially methylated regions are associated with corresponding changes in gene expression, suggesting functional relevance.

Several key observations have emerged from DNA methylation studies. First, methylation changes affect genes involved in fibrotic pathways, including those encoding extracellular matrix proteins, growth factors, and inflammatory mediators [58,59]. Second, genetic variants associated with PPF susceptibility often coincide with differentially methylated regions, suggesting interactions between genetic and epigenetic risk factors [22]. Third, environmental exposures such as cigarette smoke can influence methylation patterns, providing mechanistic links between environmental and genetic risk factors.

Specific examples of methylation-regulated genes in PPF include the tumor suppressor gene p14(ARF), which shows hypermethylation-mediated silencing in IPF lung fibroblasts [58]. This silencing contributes to fibroblast resistance to apoptosis, promoting the persistence of myofibroblasts in fibrotic lesions. Similarly, the antifibrotic gene cyclooxygenase-2 (*COX2*) demonstrates promoter hypermethylation and reduced expression in PPF lungs [58].

### 3.3. Histone Modifications

Histone modifications represent another layer of epigenetic regulation in PPF, though this area remains less well-characterized than DNA methylation [58,59]. Studies have identified altered histone acetylation and methylation patterns in PPF lungs, particularly affecting genes involved in fibroblast activation and extracellular matrix production.

The alpha-smooth muscle actin (α-SMA) gene, a key marker of myofibroblast differentiation, is regulated by DNA methylation of CpG islands in its promoter region [58]. Pharmacological inhibition of DNA methyltransferases can induce α-SMA expression in fibroblasts, while overexpression of methyltransferases suppresses expression. This regulatory mechanism directly impacts TGF-β1-induced myofibroblast differentiation, a central process in fibrotic tissue remodeling.

### 3.4. Non-Coding RNA Regulation

MicroRNAs (miRNAs) and long non-coding RNAs (lncRNAs) contribute to PPF pathogenesis through post-transcriptional regulation of gene expression [60,61]. The miR-17~92 cluster represents a particularly important example, as its promoter undergoes hypermethylation in PPF, leading to reduced miRNA expression and subsequent upregulation of profibrotic target genes [58]. This creates a feedback loop where DNA methyltransferase 1 (DNMT1) methylates the miR-17~92 promoter, and reduced miRNA expression leads to increased DNMT1 levels.

MicroRNAs (miRNAs) constitute one of the most extensively studied layers of epigenetic regulation in pulmonary fibrosis. Their dysregulation affects multiple cell types relevant to PPF, including alveolar epithelial cells, fibroblasts, macrophages, and endothelial cells [62,63].

One of the earliest and most influential discoveries was the identification of decreased expression of the *let-7* family in IPF lungs. Pandit et al. (2010) [62] demonstrated that suppression of *let-7d* promotes epithelial–mesenchymal transition, enhances TGF-β signaling, and contributes directly to aberrant epithelial plasticity. Restoration of let-7d in experimental models attenuated fibrosis, suggesting a potential therapeutic role.

Another widely characterized profibrotic miRNA is *miR-21*, which promotes fibroblast activation, inhibits SMAD7, amplifies TGF-β signaling, and correlates with disease severity in IPF. Conversely, the *miR-29* family is consistently downregulated in fibrotic lung tissue and acts as a master suppressor of extracellular matrix genes, including collagen I, collagen III, and fibrillin. Experimental delivery of miR-29 mimics reduces collagen deposition and fibrosis severity.

Additional miRNAs relevant to PPF include *miR-199a-5p*, which enhances ER stress and epithelial apoptosis; *miR-34a*, involved in cellular senescence and telomere dysfunction; and the *miR-17–92 cluster*, whose promoter is hypermethylated in PPF, leading to reduced expression and dysregulated cell-cycle control. Together, these miRNAs participate in a complex regulatory network involving TGF-β, Wnt/β-catenin, PI3K/AKT, ER-stress pathways, and fibroblast–epithelial cross-talk.

Collectively, these findings highlight miRNAs as key modulators of fibrogenesis and potential therapeutic targets. Their integration with genetic and transcriptomic profiles may further refine patient stratification and guide future precision medicine approaches in PPF.

Competitive endogenous RNA (ceRNA) networks have also been implicated in PPF pathogenesis [60]. These networks involve interactions between lncRNAs, miRNAs, and mRNAs that can modulate fibrotic gene expression. For example, the ceRNA axes KCNQ1OT1/XIST/NEAT1-miR-20a-5p-ITGB8 and XIST-miR-146b-5p/miR-31-5p-MMP16 have been associated with PPF progression [60].

## 4. Gene–Environment Interactions

The development of PPF results from complex interactions between genetic susceptibility factors and environmental exposures over time [64]. While genetic variants establish baseline risk, environmental factors often serve as triggers that initiate or accelerate disease progression in genetically susceptible individuals.

### 4.1. Environmental Risk Factors

Multiple environmental exposures have been associated with PPF development, including cigarette smoking, occupational dusts, viral infections, and air pollution [4,65]. These exposures can interact with genetic variants to modify disease risk and progression patterns. For example, cigarette smoking represents the most significant environmental risk factor for PPF, and its effects may be modified by genetic variants in genes such as *MUC5B* and *TOLLIP*.

The interaction between genetic susceptibility and environmental exposures is particularly evident in families with telomere-related variants. Individuals with telomeropathies may experience accelerated telomere shortening when exposed to environmental toxins or immunosuppressive medications [4]. This gene–environment interaction can exacerbate genetic risk and influence disease penetrance within families.

### 4.2. Occupational and Environmental Exposures

Specific occupational exposures have been linked to PPF development, including asbestos, silica, metal dusts, and organic antigens [65]. The variable responses to these exposures among individuals with similar exposure levels suggest genetic factors modulate susceptibility. Studies have identified genetic variants that influence responses to specific exposures, such as variants in antioxidant genes that affect responses to oxidative stress.

Among unaffected adults with family histories of FPF, self-reported exposures to certain metals are strongly associated with interstitial lung abnormalities on chest CT [4]. This finding suggests that genetic predisposition can sensitize individuals to environmental exposures that might be tolerated by those without genetic risk factors.

### 4.3. Implications for Disease Penetrance and Progression

Understanding gene–environment interactions has important implications for precision medicine approaches to PPF prevention and treatment [65,66]. Genetic testing could potentially identify individuals at high risk who might benefit from enhanced environmental exposure avoidance or more intensive surveillance. Additionally, genetic variants might predict responses to specific therapeutic interventions, allowing for personalized treatment selection.

The development of PRS that incorporates multiple genetic variants offers promise for risk stratification in clinical practice [67,68]. These scores can identify individuals at high risk for PPF development, potentially enabling earlier intervention and prevention strategies. When combined with environmental exposure assessments, PRS could provide comprehensive risk profiles for personalized medicine applications.

## 5. Clinical Implications of Genetic Testing

The integration of genetic testing into clinical practice for PPF has evolved rapidly, with several medical societies now providing guidance on appropriate testing strategies [7,32]. Genetic testing offers multiple clinical benefits, including diagnostic clarification, prognostic information, and risk assessment for family members.

### 5.1. Indications and Testing Strategies

Current guidelines recommend genetic testing within specific clinical scenarios [32].

Familial pulmonary fibrosis: Patients with positive family history with two or more family members affected by pulmonary fibrosis should be considered for genetic testing, as the yield of identifying pathogenic variants is high in this population.

Early-onset disease: Patients diagnosed with PPF before age 50 should need to undergo genetic evaluation, as this presentation is often associated with underlying genetic causes.

Syndromic presentations: Individuals with PPF accompanied by extrapulmonary manifestations such as bone marrow failure, liver disease, premature greying, or oculocutaneous albinism should be referred for genetic testing.

Coexistent conditions: Patients with PPF and lung adenocarcinoma should be evaluated for surfactant protein gene variants, which are associated with both conditions.

Telomere length measurement: Flow cytometry with fluorescent in situ hybridization (Flow-FISH) enables the measurement of telomere length in peripheral blood leukocytes. Short telomeres (typically < 1st percentile for age) suggest underlying telomere biology disorder and guide genetic testing strategies.

### 5.2. Genetic Testing Methodologies

**Figure 1:**
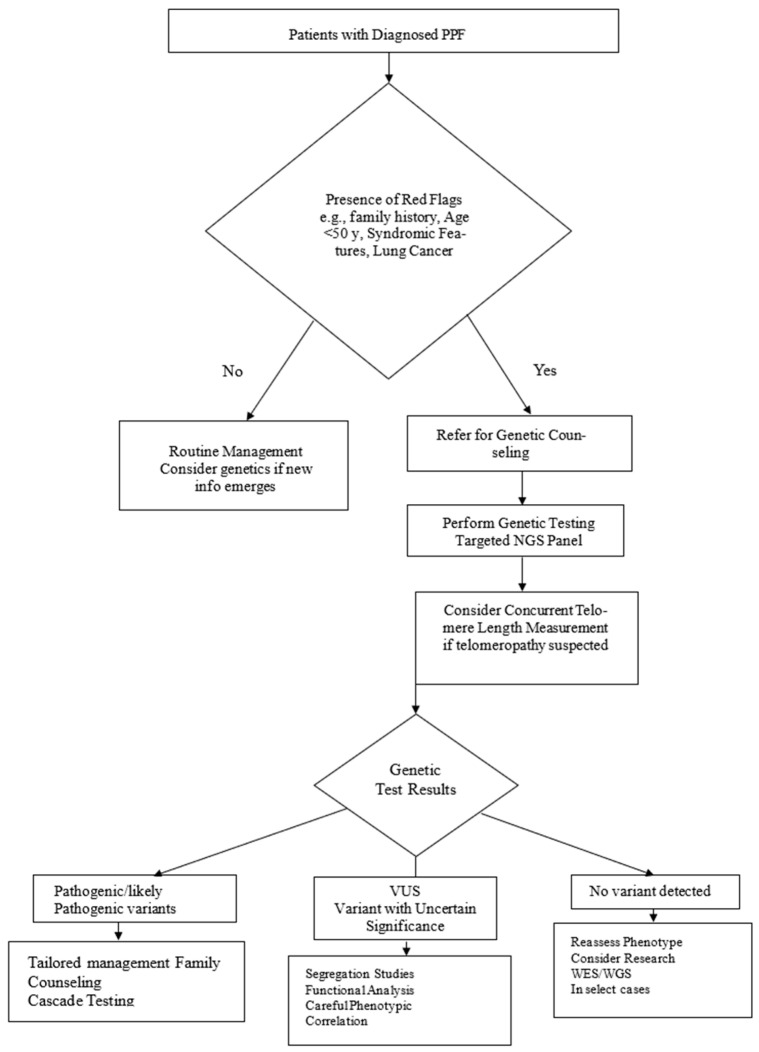
Algorithm for genetic testing in patients with progressive pulmonary fibrosis (PPF). Testing is particularly recommended for individuals with “red flag” features, such as the following: family history of ILD/PPF; early onset (<50 years); syndromic features (premature graying, cytopenias, liver dysfunction); coexisting lung adenocarcinoma; unexplained extrapulmonary manifestations. PPF—progressive pulmonary fibrosis; NGS—next-generation sequencing; VUS—variant of uncertain significance; ILD—interstitial lung disease; WES/WGS: whole-exome/whole-genome sequencing.

Clinical genetic testing for PPF typically involves two complementary approaches [32], see Figure 1.

I. Gene panel sequencing: Timely and appropriate genetic testing is essential for PPF management and risk stratification. Notably, the *MUC5B* promoter variant (rs35705950) is not included in our suggested gene panel for clinical testing. This is because rs35705950 is a common susceptibility allele with incomplete penetrance, rather than a high-penetrance pathogenic variant. Many individuals in the general population may carry the *MUC5B* risk allele without ever developing pulmonary fibrosis, so a positive test would have limited predictive value. Current clinical practice focuses on sequencing genes where mutations directly cause disease (e.g., telomerase and surfactant protein genes), and does not routinely include testing for the *MUC5B* polymorphism in diagnostic panels.

The following stepwise approach is recommended:

Step 1: Initial clinical assessment
  - Confirm diagnosis of PPF based on clinical, radiological (HRCT), and, when needed, histopathological criteria.
Step 2: Red flags for genetic evaluation
  - Family history of interstitial lung disease in ≥1 first-degree relative.

- Early-onset PPF (<50 years)
  - Syndromic features (premature hair greying, bone marrow failure, liver dysfunction, immunodeficiency).
  - Coexisting lung adenocarcinoma, especially with familial cases or in younger patients.

**Step 3: Referral for genetic counselling**
  - Discuss goals, implications, and possible results of genetic testing (e.g., pathogenic variant, variant of uncertain significance (VUS), negative result).
  - Obtain detailed family history and construct a pedigree.
**Step 4. Genetic testing strategy**
  - Perform an NGS panel covering telomere-maintenance genes (TERT, TERC, RTEL1, PARN), surfactant genes (SFTPC, SFTPA1, SFTPA2, ABCA3), and syndromic genes (HPS1, HPS3) when indicated.
  - Measure telomere length (Flow-FISH) in individuals with suspected telomeropathies.
**Step 5. Result interpretation and management decisions**
  - Pathogenic variants → tailored clinical management and family counselling.
  - VUS → segregation studies or additional phenotyping as appropriate.
  - No variant detected → reassess phenotype and exposures; consider WES/WGS in select cases.

II. Whole-exome or whole-genome sequencing: For patients in whom targeted panel sequencing is negative but clinical suspicion of genetic etiology remains high, whole-exome or whole-genome sequencing may be considered. These approaches identify rare variants outside panel genes and can detect non-coding variants, structural rearrangements, or novel candidate genes.

### 5.3. Interpretation and Clinical Management

Expertise in clinical genetics is required for the interpretation of genetic test results. Variants are classified according to established criteria as pathogenic, likely pathogenic, uncertain significance, likely benign, or benign [69]. Variants of uncertain significance (VUS) represent a particular challenge, as their clinical significance may not be clear without additional functional studies or family segregation data [69].

Positive genetic testing results have several important clinical implications [69]; the details are presented in Table 2.

### 5.4. Prognostic Information

Patients with pathogenic variants in telomere-related genes typically experience more rapid disease progression and shorter survival compared to those without such variants. This information can guide treatment decisions and transplant referral timing [42,70].

### 5.5. Surveillance Recommendations

Specific genetic variants are associated with increased risks of extrapulmonary manifestations. For example, patients with surfactant protein gene variants require enhanced lung cancer surveillance, while those with telomere-related mutations need monitoring for bone marrow failure and liver disease [71,72].

### 5.6. Treatment Considerations

Genetic results may influence treatment selection, with some studies suggesting differential responses to antifibrotic medications based on genetic background. Additionally, certain genetic variants may contraindicate specific therapies, such as immunosuppressive treatments in patients with telomeropathies [73,74].

### 5.7. Treatment Consequences and Lung Transplantation Planning

Genetic findings in progressive pulmonary fibrosis (PPF) can inform important aspects in disease management, guiding medical decisions, surveillance strategies, and lung transplant planning.

### 5.8. Telomere-Related Gene Variants (TERT, TERC, RTEL1, PARN)

#### 5.8.1. Antifibrotic Medications

The efficacy of nintedanib and pirfenidone is preserved; however, side effects may be more pronounced due to comorbidities such as cytopenias and liver dysfunction [75].

#### 5.8.2. Immunosuppression

There is an increased risk of severe cytopenia and infections among immunosuppressed people (especially after lung transplantation), which should be minimized or avoided if feasible [76,77].

#### 5.8.3. Lung Transplantation

There is a higher risk of hematologic post-transplant complications and poor wound healing after lung transplant operations; pre-transplant multidisciplinary assessment and tailored immunomodulation is advised [77].

#### 5.8.4. Malignancy, Liver, and Bone Marrow Surveillance

Regular monitoring is advisable for bone marrow failure, liver dysfunction, and malignancies, especially hematological cancers [78].

### 5.9. Surfactant Protein Gene Variants (SFTPC, SFTPA1, SFTPA2, ABCA3)

#### 5.9.1. Lung Cancer Screening

Heightened surveillance for pulmonary adenocarcinoma is advisable, especially among *SFTPA2* and *SFTPA1* variant carriers [72].

#### 5.9.2. Antifibrotic Therapy

Antifibrotics are typically indicated in treatment approaches, though their efficacy may be variable; personalized assessment is required [74].

#### 5.9.3. Early Transplant Referral

It is important to consider earlier referral for lung transplant due to possible rapid progression [79].

### 5.10. MUC5B Promoter Variant

#### Prognosis

The MUC5B Promoter variant is often associated with slower progression and improved survival, which may influence monitoring intervals [16].

### 5.11. Hermansky–Pudlak Syndrome

#### Management of Bleeding Risk

It is important to assess bleeding risk and platelet function, and avoid antiplatelet agents unless clearly indicated [80].

### 5.12. Pulmonary Fibrosis Treatment

The disease is typically characterized by rapid progression; early antifibrotic therapy and transplant planning are recommended [81].

### 5.13. Family Counselling and Cascade Testing

Family counseling: positive genetic results enable targeted testing of family members, allowing for risk stratification and early surveillance in at-risk relatives. Genetic counseling is essential to help families understand inheritance patterns, penetrance, and implications for family planning (Table 3) [82].

## 6. Genetic Biomarkers and Therapeutic Targets

The identification of genetic variants associated with PPF has led to the development of novel biomarkers and therapeutic targets. These discoveries offer potential for improved diagnostic accuracy, prognostic stratification, and personalized treatment [83].

### 6.1. Biomarkers for Diagnosis and Prognosis

Several genetic variants have demonstrated utility as biomarkers for PPF diagnosis and prognosis [68,84].

*MUC5B* promoter variant: Beyond its role in disease susceptibility, the *MUC5B* variant serves as a prognostic biomarker associated with improved survival outcomes [11,14]. This paradoxical association suggests that *MUC5B*-positive patients may represent a distinct disease endotype with different therapeutic requirements.

Telomere length: Short telomeres serve as both a biomarker of genetic risk and a prognostic indicator [42,79]. Patients with critically short telomeres typically experience more rapid disease progression and may benefit from earlier transplant evaluation.

Polygenic risk scores: incorporating multiple genetic variants can stratify individuals by disease risk and may prove useful for screening high-risk populations [67,68]. These scores achieve area under the curve values of 0.7–0.8 for PPF prediction, suggesting clinical utility.

### 6.2. Protein Biomarkers

Genetic discoveries have also led to the identification of protein biomarkers that reflect underlying pathobiological processes [68,84].

Surfactant proteins: Serum levels of surfactant proteins SP-A and SP-D are elevated in PPF patients and correlate with disease severity. These proteins may be particularly relevant in patients with surfactant protein gene variants.

Extracellular matrix proteins: Proteins such as periostin, YKL-40, and matrix metalloproteinase-7 (MMP-7) reflect ongoing fibrotic processes and may serve as biomarkers of disease activity.

Inflammatory mediators: Cytokines and chemokines including CCL-18, IL-8, and S100A4 demonstrate associations with disease progression and may be useful for monitoring treatment responses.

### 6.3. Emerging Therapeutic Strategies

Genetic insights have identified several potential therapeutic targets for PPF treatment [85,86].

#### 6.3.1. Telomerase Activation

Telomere shortening is one of the most consistent molecular hallmarks of progressive pulmonary fibrosis (PPF), particularly in patients carrying pathogenic variants in *TERT*, *TERC*, *PARN*, or *RTEL1*. Critically short telomeres lead to replicative senescence, genomic instability, and activation of profibrotic signaling in alveolar epithelial cells and fibroblasts. These insights provide a strong mechanistic rationale for therapeutic strategies aimed at telomere restoration or maintenance.

Telomerase activators—agents capable of upregulating telomerase reverse transcriptase (*TERT*) activity—have shown promise in preclinical models of telomere biology disorders. Small molecules such as danazol, a synthetic androgen, and experimental agents including TA-65 and GRN510 have demonstrated telomere elongation or stabilization in vitro and in early clinical pilot studies in related telomeropathies [87,88,89]. In the context of PPF, telomerase activation might delay epithelial cell senescence, reduce profibrotic cytokine release, and preserve alveolar integrity.

However, telomerase is also implicated in cellular immortalization and tumorigenesis. Sustained activation could potentially facilitate the clonal expansion of precancerous cells or accelerate the progression of occult malignancies, particularly in a lung environment already prone to DNA damage and chronic inflammation. For this reason, clinical development of telomerase activators is proceeding cautiously, focusing on short-term or tissue-targeted approaches and on rigorous long-term safety monitoring [88,89]. Innovative strategies under investigation include nanoparticle-based lung delivery systems and transient gene-therapy vectors designed to provide controlled, localized telomerase reconstitution without systemic exposure.

#### 6.3.2. Endoplasmic Reticulum (ER) Stress

A significant subset of PPF cases, especially those associated with *SFTPC*, *SFTPA1*, and *SFTPA2* mutations, is characterized by chronic endoplasmic reticulum (ER) stress in alveolar type II cells. Mutant surfactant proteins frequently misfold and accumulate within the ER, triggering an unfolded protein response (UPR). Persistent UPR activation induces apoptosis, epithelial injury, and profibrotic signaling cascades [90,91,92]. Therapeutic strategies that reduce ER stress or improve protein folding are therefore under intense preclinical exploration. Chemical chaperones such as 4-phenylbutyrate (4-PBA) and tauroursodeoxycholic acid (TUDCA) have shown efficacy in alleviating misfolded protein accumulation and normalizing surfactant trafficking in cellular and animal models [91,92]. Other approaches include UPR modulators, for example, small-molecule inhibitors of PERK or IRE1 pathways, which aim to fine-tune rather than completely suppress adaptive stress responses. Gene-editing techniques (e.g., CRISPR-based correction of *SFTPC* I73T mutations) are also being evaluated to correct the underlying protein defect directly.

Although these therapies remain in the preclinical or early translational stages, they represent a paradigm shift by directly addressing a root molecular mechanism of disease rather than merely slowing downstream fibrosis. The integration of ER stress biomarkers (such as BiP/GRP78 or XBP1s) into future clinical trials may facilitate patient selection and the monitoring of therapeutic responses [91,92].

#### 6.3.3. Mucociliary Clearance and Airway Mucus Hypersecretion

The common *MUC5B* promoter variant (rs35705950) remains the strongest known genetic risk factor for idiopathic and progressive pulmonary fibrosis. This gain-of-function variant drives excessive production of *MUC5B* mucin, particularly in distal airways and honeycomb cysts, potentially impairing mucociliary clearance and promoting microaspiration, chronic low-grade infection, and epithelial injury [11,17,93]. Therapeutic strategies are therefore focusing on reducing mucus accumulation or enhancing clearance. Mucolytic agents such as N-acetylcysteine (NAC), already used as antioxidant therapy, may also reduce mucus viscosity and facilitate airway clearance. Inhaled hypertonic saline or recombinant human DNase, established in cystic fibrosis, could be repurposed to improve mucus rheology in PPF. Furthermore, anti-inflammatory and mucoregulatory agents such as macrolides (e.g., azithromycin) or inhibitors of the EGFR/IL-13 axis are under consideration to reduce goblet-cell hyperplasia and mucus hypersecretion [17,93].

Beyond symptomatic relief, modulating mucociliary function may alter disease trajectory by reducing repeated epithelial injury and lowering microbial burden, as suggested by observational data linking the *MUC5B* T allele to both improved survival and distinct airway microbiota profiles [11,17].

#### 6.3.4. Inflammatory Pathways and Innate Immunity

Genetic studies implicate innate immune regulators such as *TOLLIP* (Toll-interacting protein) and *TLR*-related genes in the pathogenesis and progression of PPF. Variants like *TOLLIP* rs5743890 and rs3750920 are associated with altered lung microbiota, increased susceptibility to infection-triggered exacerbations, and differential responses to antioxidant or antifibrotic therapy [24,25,26].

These findings highlight the innate immune system as a tractable therapeutic target. Potential strategies include selective TLR antagonists or agonists to rebalance pro- and antifibrotic signaling; *TOLLIP*-mimetic molecules to dampen inappropriate TLR activation; and inhibitors of downstream pathways such as NF-κB or inflammasome components (e.g., NLRP3). Inhaled or locally delivered anti-inflammatory biologics—such as monoclonal antibodies against IL-1β or IL-18—are also under investigation [24,26].

Importantly, a genetically guided approach is emerging: stratifying patients according to *TOLLIP* or TLR polymorphisms may assist in identifying those most likely to benefit from specific immune modulators, reducing the risk of broad immunosuppression and its associated infections. Integration of genomic and microbiome data in future clinical trials will be critical to refine these precision immunomodulatory therapies [24,25,26].

## 7. Future Perspectives and Precision Medicine

Looking ahead, continued integration of genomic, epigenomic, and transcriptomic data will accelerate the shift toward precision medicine in PPF. Emerging tools such as machine-learning-based risk prediction, single-cell multi-omics, and high-throughput functional assays are expected to be used in refining disease subtyping, improving early detection, and guiding individualized treatment selection. Increasing the availability of genetic testing may also support earlier referrals for antifibrotic therapy or lung transplantation, while enabling targeted surveillance for extrapulmonary complications in telomere or surfactant-related syndromes. Ultimately, improved understanding of the genetic architecture of PPF raises the possibility that disease progression could be predicted and potentially prevented through more personalized monitoring and intervention strategies.

Genomic discoveries are reshaping how PPF is diagnosed, stratified, and treated, establishing a foundation for increasingly individualized care across the spectrum of fibrotic lung disease.

## 8. Conclusions

Progressive pulmonary fibrosis genetics has been exceedingly clarified over the past two decades, unraveling a complex architecture including common and rare gene variants. The finding of seminal genes such as *MUC5B*, telomere-related genes, and surfactant protein genes has provided fundamental insight into pathogenesis of the disease and has disclosed novel therapeutic development avenues.

Clinical integration of genetic testing has already begun to transform PPF care so that more accurate diagnosis, classification in terms of prognosis, and counseling for families are possible. As our understanding of genetic risk factors is expanded, the potential of personalized medicine approaches increasingly becomes a reality. Developments in polygenic risk scores, treatment algorithms guided by biomarkers, and gene-based therapies based on molecular profiles hold the potential to provide increasing improvements in results among patients with this devastating disease.

Future research will likely focus on expanding genetic discovery through larger and more diverse populations, integrating multi-omics data for identifying disease phenotypes, and developing personalized treatment approaches based on genetic profiles. The goal is to transform PPF from a uniformly fatal disease to one where outcomes could be predicted and thus potentially prevented through precision medicine approaches.

The genetic revolution in PPF research has already yielded significant clinical benefits and offers hope for continued progress. As we continue to unravel the genetic determinants of this complex disease, we move closer to the goal of personalized medicine that can improve outcomes for patients and their families affected by progressive pulmonary fibrosis.

## Figures and Tables

**Table 1:** Key genes and loci associated with progressive pulmonary fibrosis (PPF).

Gene/Locus	Biological Pathway/Function	Variant Type	Key Clinical Associations/Implications
*MUC5B*	Mucin production, mucociliary clearance	Common promoter variant (rs35705950)	- Strongest genetic risk factor (~6–20× increased risk). - Paradoxically associated with slower progression and better survival. - Population stratification (common in European populations).
*TERT*, *TERC*, *PARN*, *RTEL1*	Telomere maintenance	Rare deleterious variants and common SNPs	- Most common cause of familial PF (~15–20% of FPF). - Associated with rapid progression, shorter survival, extrapulmonary manifestations (bone marrow, liver). - Short telomeres are a key biomarker.
*SFTPC*, *SFTPA1*, *SFTPA2*, *ABCA3*	Surfactant metabolism and homeostasis	Rare missense/loss-of-function variants	- Often early-onset disease. - Associated with lung adenocarcinoma (*SFTPA1*/*A2*). - Histologic heterogeneity (UIP, NSIP, DIP).
*DSP* (Desmoplakin)	Cell adhesion, epithelial integrity	Common SNP (rs2076295)	- Associated with disease risk and differential response to antifibrotics (better response to nintedanib in G allele carriers).
*TOLLIP*	Innate immunity, TLR signaling	Common SNPs (rs5743890, rs3750920)	- rs5743890: associated with faster progression. - rs3750920: potential pharmacogenetic interaction with N-acetylcysteine.
*FAM13A*	Wnt/β-catenin signaling, possibly (function not fully defined)	Common SNP (rs2609255)	- Influences susceptibility across populations and ILD subtypes (IPF, RA-ILD).
*KIF15*, *MAD1L1*	Mitotic spindle assembly, cell cycle regulation	Common SNPs and rare variants	- *KIF15*: associated with early-onset, severe disease. - *MAD1L1*: consistent risk locus, specific phenotype correlations not yet defined.
*DEPTOR*	mTOR signaling inhibition	Common SNP	- Decreased expression increases risk. Associated with brain structural changes, potentially linking to neuropsychiatric comorbidities.
*HPS1*, *HPS3*, *HPS4*	Lysosome-related organelle biogenesis (Hermansky–Pudlak Syndrome)	Rare loss-of-function variants	- Syndromic PF with oculocutaneous albinism, bleeding diathesis. - Aggressive, early-onset PF (especially *HPS1* and *HPS4*).

**Table 2:** Clinical implications of major genetic variants associated with PPF.

Genetic Finding	Key Clinical Implications
Telomere-related gene variants (*TERT*, *TERC*, *RTEL1*, *PARN*)	- Use antifibrotic therapy with caution; monitor closely for cytopenias and liver dysfunction (higher drug toxicity due to telomere biology defects). - Avoid or minimize immunosuppression because of increased risk of bone marrow failure and infections. - Lung transplant candidates require tailored peri-transplant immunosuppression due to higher complication rates. - Enhanced surveillance for bone marrow dysfunction, liver disease, and hematologic malignancies.
Surfactant protein gene variants (*SFTPC*, *SFTPA1*, *SFTPA2*, *ABCA3*)	- Implement enhanced lung cancer screening, especially for *SFTPA1*/*SFTPA2* carriers (higher lung adenocarcinoma risk). - Antifibrotic therapy is indicated, but treatment response may vary—monitor progression closely. - Consider earlier referral for lung transplantation, as disease often progresses rapidly at younger ages.
MUC5B promoter variant (rs35705950)	- Carriers often have a slower disease trajectory (better survival, slower FVC decline). - In stable patients, follow-up intervals may be spaced slightly more. - Standard antifibrotic treatment remains appropriate based on clinical status.
Hermansky–Pudlak syndrome genes (*HPS1*, *HPS3*, *HPS4*)	- Manage bleeding risk proactively; avoid anticoagulants/antiplatelets unless essential. - Pulmonary fibrosis in HPS is often aggressive → treat like IPF: early antifibrotic therapy and early transplant referral. - Requires multidisciplinary care, especially hematology involvement.
DSP variant (rs2076295, desmoplakin)	- Variant influences prognosis and may guide antifibrotic choice. - G allele: better response to nintedanib. - TT genotype: may respond better to pirfenidone. - Represents a potential pharmacogenomic biomarker (confirmation needed).
TOLLIP variants (rs5743890, rs3750920)	- rs5743890 minor allele: poorer prognosis (shorter survival, faster progression). - rs3750920 T allele: historical evidence of better response to N-acetylcysteine (NAC) (pre-antifibrotic era). - Useful as prognostic and potential predictive markers.

**Table 3:** Summary of the clinical management recommendations based on genetic findings in progressive pulmonary fibrosis (PPF).

Genetic Finding	Key Management Considerations
Telomere-related gene variants (*TERT*, *TERC*, *RTEL1*, *PARN*)	Antifibrotics: Use with caution; monitor for side effects (cytopenias, liver dysfunction). Immunosuppression: Generally avoid/minimize due to high risk of cytopenias and infections. Transplant: Higher peri- and post-transplant risks; requires multidisciplinary assessment and tailored immunosuppression. Surveillance: Regular monitoring for bone marrow failure, liver disease, and hematological malignancies.
Surfactant protein gene variants (i.e., *SFTPC*, *SFTPA1*, *SFTPA2*, *ABCA3*)	Cancer screening: Enhanced surveillance for lung adenocarcinoma (especially for *SFTPA1*/*A2*). Antifibrotics: Indicated, but efficacy may be variable; personalized assessment needed. Transplant: Consider earlier referral due to potential for rapid progression.
*MUC5B* promoter variant (rs35705950)	Prognosis: Often indicates a slower disease trajectory; may allow for less frequent monitoring in stable patients.
Hermansky–Pudlak Syndrome (i.e., *HPS1*, *HPS3*, *HPS4*)	Bleeding risk: Manage bleeding diathesis; avoid antiplatelet agents unless essential. PF Management: Disease is often aggressive; early antifibrotic therapy and transplant planning are recommended.
*DSP* rs2076295	Treatment selection: G allele carriers may respond better to nintedanib; TT homozygotes may benefit more from pirfenidone (preliminary evidence).
*TOLLIP* variants	Prognosis and therapy: rs5743890 minor allele may indicate poorer prognosis. rs3750920 may predict response to N-acetylcysteine (historical context).

## Data Availability

No new data were created or analyzed in this study. Data sharing is not applicable to this article.

## Abbreviations

The following abbreviations are used in this manuscript:

PPF	progressive pulmonary fibrosis
IPF	idiopathic pulmonary fibrosis
GWAS	genome-wide association studies
TGF-β	transforming growth factor-β
VUS	variants of uncertain significance
PRS	polygenic risk scores
MMP-7	matrix metalloproteinase-7
FPF	familial pulmonary fibrosis
DNA	deoxyribonucleic acid
RNA	ribonucleic acid
MAF	minor allele frequency
FVC	forced vital capacity
DLCO	Diffusing Capacity of the Lungs for Carbon Monoxide
mTOR	mammalian target of rapamycin
NSIP	nonspecific interstitial pneumonia
DIP	desquamative interstitial pneumonia
UIP	usual interstitial pneumonia
ILD	interstitial lung disease
CT	computed tomography
HRCT	high-resolution computed tomography
SNP	single-nucleotide polymorphisms
TLR	Toll-like receptor
FISH	fluorescent in situ hybridization
NGS	next-generation sequencing
WES/WGS	whole-exome/whole-genome sequencing
ER	endoplasmic reticulum
RA-ILD	rheumatoid-arthritis-associated interstitial lung disease
